# Estimated reductions in type 2 diabetes burden through nutrition policies in AZAR cohort population: A PRIME microsimulation study for primary health care

**DOI:** 10.34172/hpp.42452

**Published:** 2024-03-14

**Authors:** Samira Pourmoradian, Naser Kalantari, Hassan Eini-Zinab, Alireza Ostadrahimi, Jafar Sadegh Tabrizi, Elnaz Faramarzi

**Affiliations:** ^1^Nutrition Research Center, Department of Community Nutrition, Faculty of Nutrition and Food Sciences, Tabriz University of Medical Sciences, Tabriz, Iran; ^2^Department of Community Nutrition, National Nutrition and Food Technology Research Institute, Faculty of Nutrition Sciences and Food Technology, Shahid Beheshti University of Medical Sciences, Tehran, Iran; ^3^Nutrition Research Center, Tabriz University of Medical Sciences, Tabriz, Iran; ^4^Department of Health Service Management, Tabriz Health Service Management Research Centre, School of Health Management and Medical Informatics, Tabriz University of Medical Sciences, Tabriz, Iran; ^5^Liver and Gastrointestinal Diseases Research Center, Tabriz University of Medical Sciences, Tabriz, Iran

**Keywords:** Diabetes mellitus, Type 2, Sugar-sweetened beverages, Health policy

## Abstract

**Background:**

Given the impact of high intake of sugar-sweetened beverages on type 2 diabetes, intervention to reduce their consumption can be a top priority for any health system. Thus, the purpose of the present study is to simulate the impact of policy options related to reduce consumption of sugar-sweetened beverages (SSBs) on the prevalence and mortality of type 2 diabetes in Iranian men and women.

**Methods:**

A discrete event simulation (DES) model was used to predict the effect of several policy options on the prevalence and death from type 2 diabetes in Azar Cohort Databases. Population age- and sex-specific prevalence and incidence rate of diagnosed diabetes were derived from the national health data. The Preventable Risk Integrated Model (PRIME) model was used for coding the input parameters of simulation using R and Python software.

**Results:**

The prevalence and mortality rate of type 2 diabetes under the scenario of reduced consumption of SSBs indicated that the highest and the lowest prevalence and mortality rates of type 2 diabetes for men and women were related to no policy condition and replacing SSBs with healthy drinks, like water, respectively. Also, the maximum "number of deaths postponed/ prevented" from type 2 diabetes was related to replacing SSBs with water (n=2015), and an integration of reformulation and applying 10% tax on SSBs (n=1872), respectively.

**Conclusion:**

Simulating the effect of different policy options on reducing the consumption of SSBs showed "replacing of SSBs with water" as the most effective policy option in Iranian setting.

## Introduction

 Diabetes mellitus (DM) is a highly prevalent leading cause of mortality in the Middle East and North Africa (MENA) region. It is estimated that about 35 million people are living with diabetes in this region. Iran is amongst the countries of the region with the highest prevalence of DM (8.9% in adult population).^1^

 Evidence suggest that sugar-sweetened beverages (SSBs) consumption may increase the risk of obesity and type 2 diabetes through increasing body weight, impairing ß-cell function, and promoting hepatic lipogenesis and insulin resistance.^2-4^

 To reduce SSBs consumption and to prevent diet-related non-communicable diseases (NCDs), several upstream and downstream interventions have been globally proposed, including improving consumer information, taxation, and reformulation of SSBs and so on.^5^ However, the effects of these policy options on the incidence and mortality of type 2 diabetes are broad and potentially confusing.

 To investigate the lack of sufficient perspectives on the impact of various policies on reducing the nutritional risk factors of NCDs, some statistical methods, such as simulation, may be used. Computer simulation is a powerful tool to inform policymakers about the consequences, strengths, and weaknesses of different policy options before implementation. Nowadays, these methods are used in different countries as one of the most basic methods of macro-decision-making and policy-making.^6-8^

 To the best of our knowledge, no modeling study has compared the effects of several nutritional policies on reducing SSBs consumption, aiming at decreasing the prevalence and mortality rate of type 2 diabetes. Accordingly, this study aimed (1) to quantify the differences in the prevalence of type 2 diabetes, and (2) to compare the number of deaths from type 2 diabetes which could be averted among the implementation of various nutrition policy options for reducing SSBs consumption and no policy implementation from 2015 to 2035 in Iranian population.

## Materials and Methods

 This study used a discrete event simulation (DES) model, the Preventable Risk Integrated Model (PRIME),^9^ to estimate age-specific and gender-specific changes in the prevalence and mortality of type 2 diabetes that would be resulted from the changes in SSBs intake in the Azar cohort population. The reported procedures were conducted based on the International Society for Pharmacoeconomics and the Outcomes Research-Society for Medical Decision Making (ISPOR-SMDM) guidelines for modelling research. We also applied the generic reporting checklist for healthcare-related DES studies developed by Zhang et al.^10^

###  The Preventable Risk Integrated model 

 The PRIME model is a scenario-based model that links 12 behavioral risk factors, such as diet, physical inactivity, alcohol consumption and tobacco consumption, to NCDs mortality.^11^ This model has 24 health outcomes, including cardiovascular diseases, cancers, kidney disease, liver disease, chronic obstructive pulmonary disease, and type 2 diabetes (see Supplementary file 1, Figure A).

###  Iran-specific epidemiologic input

####  AZAR cohort data 

 This study used the data from the Azar Cohort (https://azarcohort.tbzmed.ac.ir/) study, which is a section of the Prospective Epidemiological Research Studies in Iran (Persian cohort),^12^ started in 2015 in Shabestar county, East Azerbaijan province, Northwest of Iran (see Supplementary file 1).

###  Incidence rate and mortality from type 2 diabetes in Iran

 According to the study conducted by Derakhshan et al, the annual gender-specific incidence rate of type 2 diabetes in Iranian men and women were 9.36 and 10.1 per 1000 persons, respectively.^13^ The mortality rate of type 2 diabetes in the study population in 2015 was based on the data from the National and Subnational Burden of Diseases, Injuries, and Risk Factors (NASBOD) in Iran.^14^

###  Base case sugar-sweetened beverage consumption in Iran

 Data from the Azar cohort study, were used to estimate the daily SSBs intake. A Food Frequency Questionnaire (FFQ) assessment administered to all AZAR cohort participants to assess the amount and frequency of food and drink consumption over the past year. The SSBs were defined as any sugar-sweetened sodas, fruit drinks, or homemade SSBs, which contained at least 50 kcal per 8-oz serving, with 100% fruit juice being excluded.According to the study of Malik et al, 236 mL of SSBs was consider as one serving of SSB.^3^

###  Effects of sugar-sweetened beverage on type 2 diabetes 

 Based on the review of epidemiological studies, we modeled direct and indirect effects of SSBs consumption on DM. We considered a meta-analysis of 8 prospective cohorts with a total of 310 819 participants and 15043 cases of type 2 DM. In this meta-analysis, the individuals in the highest category of SSBs intake (1–2 servings/d) had a 26% greater risk of developing type 2 DM, compared to those in the lowest category of SSBs intake (none or <1 serving per month; risk ratio, 1.26; 95% CI,1.12–1.41). In this meta-analysis study, the reported RR was adjusted for energy intake. The association between SSBs intake and the risk of type 2 DM in this meta-analysis was consistent across gender and ethnic groups, which included blacks, whites, and Asians. Although there was heterogeneity across the studies (I^2^ = 66%), all but one showed positive associations between SSBs intake and the risk of type 2 DM. Also, the strength of the association was increasing by increase in the study size and duration.^3^

###  Model simulation

 We applied the PRIME to predict the impact of reduction in SSBs consumption on incidence, mortality, and the number of deaths postponed or prevented (DPP) from type 2 diabetes over 20 years (2015-2035) under several policy scenarios. According to our earlier report, we selected the highest priority policy options (in the view of Iranian health and nutrition policy stakeholders related to reducing the burden of NCDs in Iran) for simulation.^15^ These policy options were applying 10% tax on SSBs, replacing SSBs with water, the reformulation of SSBs through reducing the sugar content of SSBs by 30%, and an integration of both tax and reformulation policies. These policy options were described in detail in the supplementary file. However, the “no policy scenario” was also simulated assuming that the current situation is maintained and no special policy is implemented. According to the methodology of a similar study to obtain DPP in the case of implementing a policy option in an age and gender group, the desired command code was written and implemented in the software (see Supplementary file 1).

###  Sensitivity and uncertainty analyses

 The PRIME model has an in-built with Monte Carlo simulation to quantify 95% uncertainty intervals (UIs) in the attributable deaths from SSBs intake data (which includes both measurement and sampling errors and modeling uncertainty), and uncertainty from the relative risks in our final estimates that have been derived from dose-response meta-analysis studies. These intervals are representative of uncertainty in the model parameters, and are not related to the variability of the original data used in the baseline and policy options.

 Each policy scenario was simulated 1000 times for generating 95% CIs for each of the estimates of the model outputs under the counterfactual scenario. For each mean exposure, population-representative standard deviations were predicted using coefficients from regressions performed on all available dietary survey data in our collection. The standard deviation was the dependent variable while the mean was the independent variable.^16,17^ All analyses were performed using R and Python software, version 2.15.0.

###  Model validation

 The model structure, assumptions, all input data sources, and findings were sent to an expert panel comprising statisticians and health and nutrition professionals to compare with real world circumstances, and to assess the face validity of the model. To determine internal validity, a sensitivity analysis was carried out.

## Results

 The data of 12 126 participants from Azar cohort study were used. General characteristics of the population including age, gender, marital status, education, and SSBs consumption are reported in Table 1. The impact of policy options on reducing the consumption of SSBs has been simulated in the Azar cohort population. In total, 57.1% of the studied population were women, and 92.40% were married. Also, 18.78% of the study population were illiterate, 50.15% had primary literacy, 12.94% was middle school education, 10.4% had a diploma degree, and only 7.73% had university education. At the time of data collection in 2015, about 11.65% had type 2 diabetes, with a prevalence of 12.51% in women and 10.50% in men (Table 1).

**Table 1:** Baseline demographic characteristics of study population

**Variable **	**Male ** **(n=5205)**	**Female ** **(n=6921)**	**Total ** **(n=12126)**
Age (y)^*^	50.25 ± 9.25	48.92 ± 9.26	49.53 ± 9.28
Education level (frequency)			
Illiterate	1291	3075	4366
Elementary	1875	2119	3994
Mid school	825	744	1569
High school graduated	769	747	1516
University education	445	236	681
Marital status			
Single	59	862	921
Married	5146	6059	11205
Type 2 diabetic patients (frequency)	547	866	1413
Sugar sweetened beverages daily consumption^*^ (mL/day)	62.88 ± 8.29	38.57 ± 7.35	49.00 ± 8.1

*The results were expressed as mean ± standard deviation.

 The effects of simulating different policy options on the prevalence of type 2 diabetes are presented in Table 2. Comparing the different simulated policy options, we identified that the “No policy scenario” was associated with the highest prevalence of diabetes in both men and women. In the other hand, “replacing SSBs with water” and “applying 10% tax on these drinks”, along with “reformulation of SSBs” had the greatest effect on reducing the prevalence of type 2 diabetes in both men and women. “Applying 10% tax” had the minimum effect on reducing the prevalence of type 2 diabetes.

**Table 2:** Gender differences in the estimated number and prevalence of type 2 diabetes during 20 years under different policy scenarios

**Variables**	**2025**				**2035**			
	**Female**		**Male**		**Female**		**Male**	
Policy scenario	**n**	**Prevalence (%)**	**n**	**Prevalence (%)**	**n**	**Prevalence (%)**	**n**	**Prevalence (%)**
No policy	1538	22	997	18	1783	26	1334	24
10% tax of SSB	1431	21	931	17	1659	24	1243	23
Reformulation	1111	16	712	13	1428	21	1003	19
Water substitution	857	12	548	10	953	14	626	13
Integrated reformulation and taxation	1001	14	651	12	1163	17	868	16

**Figure 1:**
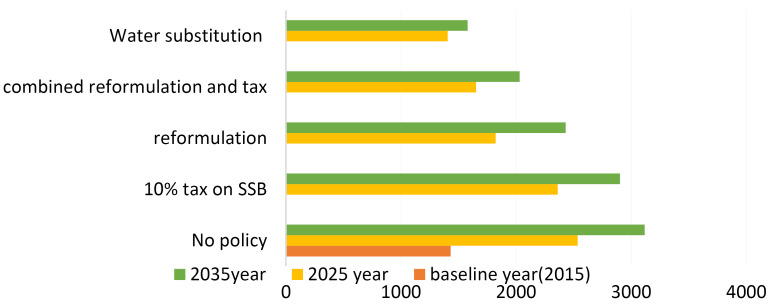
Number of patients with type 2 diabetes during 2015 until 2035 for each policy modelled

 The simulation of different policy options for reducing the consumption of SSBs on the mortality associated with type 2 diabetes over 20 years indicated that for women the “no policy option” would be resulted in 7 and 11 type 2 diabetes-associated deaths, and for men the policy would be resulted in 7 and 8 the disease-associated deaths. The lowest mortality rate from type 2 diabetes was related to implementing the “replacing SSBs with water” policy option. In women, type 2 diabetes-associated mortality rate was more affected by the implementation/non-implementation of the policy options, compared to men (Table 3, Figure 1).

**Table 3:** Gender differences in the estimated number of deaths prevented under different policy scenarios, during 20 years

**Variables**	**2015 until 2025**				**2026 until 2035**				**Total**
	**Female**		**Male**		**Female**		**Male**		
**Policy scenario**	**n (95% UI)**	**Death rate per 1000**	**n (95% UI)**	**Death rate per 1000**	**n (95% UI)**	**Death rate per 1000**	**n (95% UI)**	**Death rate per 1000**	**DPP**	**DPP/10** ^ 5 ^
No policy	314 (282,391)	8	305 (252,376)	7	953 (881,983)	11	858 (785,890)	9	-	-
10% tax of SSB	189 (176,201)	5	172 (163,180)	6	111 (100,132)	8	128 (98,145)	7	1599	435
Reformulation	170 (158,182)	4	166 (155,179)	5	97 (79,107)	6	107 (87,125)	6	1789	487
Water substitution	143 (128,151)	3	134 (119, 142)	2	76 (59,86)	4	62 (49,75)	3	2015	548
Integrated reformulation and taxation	158 (134,170)	4	141 (125, 159)	3	102 (83,124)	5	87 (93,69)	4	1872	510

UI, uncertainty interval; DPP, Death prevented/postponed.

**Figure 2:**
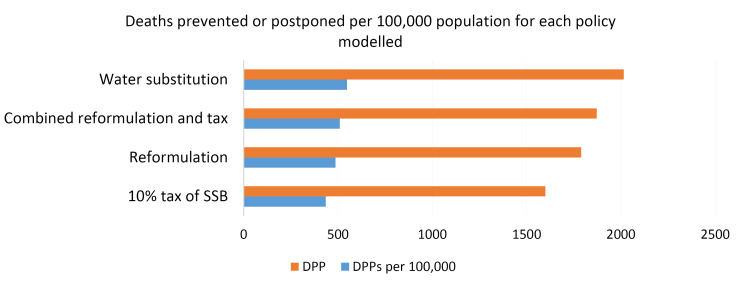
Deaths prevented or postponed per 100 000 population for each policy modelled

 The results of simulating policy options on DPP from type 2 diabetes showed that the lowest (1599) and the highest (2015) DPP levels were associated to “levying 10% tax on SSBs” and “replacing SSBs by water”, respectively (Figure 2).

## Discussion

 The results of different policy options simulations on the prevalence and mortality from type 2 diabetes showed that the “replacement of SSBs with water” over 20 years would lead to the greatest reduction in the prevalence and mortality of type 2 diabetes. However, the “No policy option” would lead to an increase in the prevalence and mortality from type 2 diabetes across the studied population. The lowest and the highest numbers of deaths prevented/postponed were related to “10% taxation” (1599 death cases) and “SSBs replacement with water” (2015 death cases), respectively. Also, “reformulating of SSBs” over 20 years can prevent 1789 deaths caused by type 2 diabetes.

 Previous studies have shown that SSBs consumption can increase the risk of NCDs, such as type 2 diabetes. In this regard, one of the effectiveness proposed strategies is to replace sugary drinks with water in preventing obesity and type 2 diabetes.^18^

 Evidence suggests that “replacing SSBs with water, low-calorie drinks, or milk” has a positive effect on reducing their consumption.^19^ “Replacing SSBs with milk” instead of non-calorie drinks, such as water, can be helpful in providing minerals and vitamins to poor and unhealthy diets. The potential effects of milk on bone health are also obvious, and daily consumption is associated with a reduction in body fat and/or body mass index. Further, the milk protein may increase lean body mass of individuals.^5,20^ Consistent with the findings of the present study, Stookey et al also found that substituting calories in SSBs with water could help people in losing weight, and consequently reducing their risk of developing chronic diseases, such as diabetes.^21^

 It is noteworthy that the findings of the effect of “replacing SSBs with water” on weight loss and thus diabetes prevention are contradictory. For example, Tate et al reported that the replacement of caloric beverages with non-caloric beverages, as a weight-loss strategy resulted in average of 2 to 2.5% weight losses.^22^ In the studies conducted by Pan et al and Piernas et al, “replacing SSBs with water” led to a significant increase in fruit and vegetable consumption, and also an improvement in the biomarkers associated to type 2 diabetes, such as fasting blood glucose and improved hydration.^23,24^ In contrast, Ebbeling et al. indicated that home delivery of low calorie beverages (e.g., bottled water and “diet” beverages) during 25 weeks resulted in an insignificant net difference in body mass index between intervention and control groups. However, they argued that several obesity related factors including group differences in television viewing hours and dietary quality, may have affected their results.^25^ In another report, this strategy resulted in a 12% reduction in SSBs consumption and 4% increase in the consumption of other beverages, such as water, which was in accordance with our results.^26^

 “Replacing SSBs with water” seems to be an optimistic scenario. However, it seems to be relatively impossible to completely replace SSBs with water in the real world. Meanwhile, evidence have shown that success can be achieved by providing individuals with education on nutrition and counselling, and especially improving access to water, through family-based interventions, like sending free water to houses. A review study has also concluded that educational interventions and the provision of water to increase water consumption within families through the strategies like reducing water price might be successful in reducing SSBs consumption.^27^ In a British study, it was estimated that the partly replacement of SSBs with water, tea or coffee could reduce the incidence of type 2 diabetes by 14 to 25%.^28^

 The reformulation of SSBs to reduce the energy density of the beverages per serving is one of the proposed policies of different countries to reduce SSBs consumption. As part of the UK’s public health contract, the mandatory reformulation of SSBs to reduce their sugar and energy density is the most obvious example of this scenario. This program was launched in 2012 by the UK Department of Health to involve the food and beverages industries in helping people to reduce their calorie intake. In a modeling study in Australia, the reformulation of SSBs was reported to be one of the most cost-effective policies in the prevention of NCDs.^29^ As far as our current knowledge, our study was the first in comparing the effectiveness of this policy option with others to reduce the prevalence and the number of deaths associated to type 2 diabetes. Based on our findings, compared with no policy and taxation scenarios, the reformulation policy had a greater impact on reducing the prevalence and mortality associated to type 2 diabetes.

 In our study, in comparison with other policy scenarios (except for “no policy scenario”), applying a 10% taxation on SSBs had a minimum effect on reducing the incidence and mortality associated to type 2 diabetes. Fiscal tools, such as taxation, are other common policies to be applied to control unhealthy food consumption. Mexico and New Zealand are the leading countries in levying tax on SSBs, which have been shown to be relatively successful in limiting SSBs consumption.^30^ A modeling study in Mexican adults estimated that a 10% elevation in SSBs prices from 2013 to 2022 would reduce 189300 cases of type 2 diabetes and save $ 983 million in Mexico.^31^ In Australia, the taxation could save 112 000 and 56 000 years of healthy living for men and women, respectively, which could also lower the total cost of health care by 609 million dollar. Indeed, this policy would reduce the number of new cases of type 2 diabetes by about 800 people per year. Twenty-five years after the introduction of the taxation, there would be 4400 fewer cases of heart diseases and 1100 fewer cases with stroke. The taxation could also generate about $ 400 million in revenue each year.^32^ Moreover, the results of a meta-analysis study showed that “applying a 10% tax on unhealthy foods” can reduce the consumption of these foods by an average of 7%.^33^

 According to previous studies, the tax on SSBs in the populations with low socioeconomic status may lead to a further reduction in the purchase or consumption of SSBs. Backholer et al reported that applying 10% tax on SSBs can lead to a 3%-10% reduction in SSBs consumption, which varied across different social and economic classes of people consuming these beverages.^34^ A strength of our study was to consider this issue in the respondents, based on contextual indicators such as education level, income, employment in different social and economic groups. We ultimately identified that 10% tax on SSBs would prevent 1599 deaths from type 2 diabetes in 20 years. In some previous studies, experts have suggested that the tax on SSBs should be 20% or higher to have a significant effect.^35,36^ Alternatively, it should be combined with other policy interventions. In our study, the combination of taxation with reformulation of SSBs reduced 1872 deaths from type 2 diabetes. In previous studies, the mechanism of the effect of SSBs taxation on reducing the prevalence and mortality of diabetes has been discussed. The most remarkable mechanism is “reducing calorie intake” and thus “encouraging weight loss”, which consequently prevents type 2 diabetes and increases life expectancy.^33^

 In parallel with our results, previous studies indicated that fiscal policy alone was not effective in reducing SSBs consumption.^37,38^ As noted, the mortality rate of type 2 diabetes in the case of implementation/non-implementation of policy options over a 20-year period was generally higher for women than men. In consistent with our findings, Papier et al showed a significant relationship between the rate of type 2 diabetes and SSBs consumption in women, but not in men.^17^ Other studies in African, Caucasian, and Asian populations have also shown significant associations between SSBs consumption and an increased risk of type 2 diabetes in women, but not in men.^16,39,40^

 The current studies have several strengths. To the best of our knowledge, this is the first modeling study predicting the impacts of several nutritional policies on burden of type 2diabetes in Iran. Furthermore, our model predicts type 2 diabetes burden using high-quality epidemiologic studies as model inputs and cohort study as a basic population. In this research for increase validity of model each scenario was simulated 1000 times.

 As with all studies, a number of limitations need to be pointed out. Firstly, forecasting the number of deaths from type 2 diabetes over the next 20 years might be accompanied by uncertainty. One source of uncertainty arises from model input data as well as the fixed age- and gender specific prevalence, incidence and mortality risk rates over 20 years, which all were assumed based on Markov chain model. Another limitation to be considered was that some socio-economic factors such as food insecurity as a result of climate change^41^ and fiscal policies affect population real food intakes, on the other hand, change in physical activity level, implementation of screening policies to detect pre diabetes cases were affected type 2 diabetes incidence and mortality rate during the years. In addition, due to scarcity of information, changes in economic, and healthcare system characteristics over time were not included in the model.

## Conclusion

 To the best of our knowledge, the present study was the first study to simulate the impact of nutritional policy options on the incidence and mortality rate of type 2 diabetes in Iran, which could anticipate the effectiveness of the policies prior to their implementation. Our study also used the “number of deaths postponed/prevented” variable, which is more practical and understandable than other cost-effectiveness indicators for health policy makers.^42^

## Acknowledgements

 We appreciate the contribution by investigators and staff of the Azar Cohort Study. This article was extracted from Samira Pourmoradian PhD dissertation.

## Competing Interests

 The authors declare that they have no competing interests.

## Ethical Approval

 This study was approved by Ethics Committee in Shahid Beheshti University of Medical Sciences (Ethics No. IR.SBMU. NNFTRI.1397.056).

## Supplementary Files

 The supplementary file provides detailed methodology information, including model rules, assumptions, and database characteristics.
