# Supplementary material for: Estimated reductions in type 2 diabetes burden through nutrition policies in AZAR cohort population: A PRIME microsimulation study for primary health care
Source: Health Promot Perspect. 2024 Mar 14;14(1):53–60. doi: 10.34172/hpp.42452 (PMC11016142; doi:10.34172/hpp.42452)
Supplement: Supplementary file 1 — The supplementary file provides detailed methodology information, including model rules, assumptions, and database characteristics. [file hpp-14-53-s001.pdf]

Pourmoradian et al, **Health Promotion Perspectives**, 2024, 14(1), 53-S1
doi: 10.34172/hpp.42452
https://hpp.tbzmed.ac.ir

1-The Model Description

This framework is parameterized using the best available evidence from meta-analyses of epidemiological studies: prospective cohort studies for links dietary risk factor with NCD mortality[1, 2]. PRIME requires data on prevalence of modifiable risk factors, mortality rates, and population estimates by 5-year age groups and sex, for both baseline and counterfactual scenarios. For this study, PRIME was used to estimate the incidence, prevalence, number and death rate of type2 diabetes that would be delayed or averted in the studied population. The baseline scenario (no policy option) used the current levels of modifiable risk factors in those living in areas, where the counterfactual scenario involved applying the level of risk factors (Figure A).

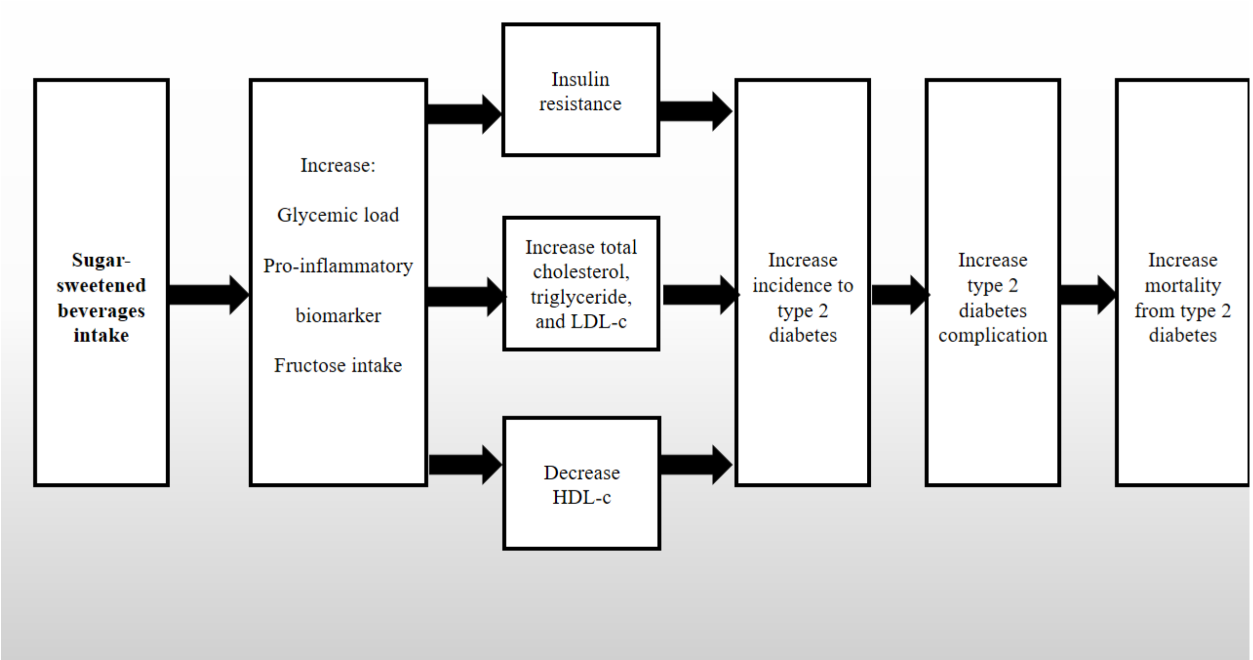

Figure A- the PRIME model

2-Model assumptions

1- It is assumed that people's behavior, including dietary intakes, has a steady trend over time.

2. With each year of progress in simulation, one year is added to the age of the subjects.

3- Based on the normal mortality rate of the population, a number of people in the basic population would die randomly and leave the model.

4- To maintain the dynamics of the model based on population growth rate and population age pyramid composition tables, a number of samples are added to the base population sample annually.

5. Based on the incidence rate of the type 2 diabetes, every year a number of healthy people randomly develop type 2 diabetes.

6- Based on the mortality rate due to type 2 diabetes, a number of people with type 2 diabetes die randomly every year.

7. The changes between the amount of before and after simulation consumption have the same distribution in the whole population (the rate of increase or decrease in consumption occurs in the same population of the study population).

8. The probabilities of the transition from one year to the next follow the rules of the Markov chain, which means that the probability of the next transition depends only on the current situation and not the previous one.

9. The future incidence and mortality rate held constant into the future.

Table A. Data sources used for type 2 diabetes PRIME Model- Iran inputs	
**Variable**	**Data Sources**
Population 2015	Azar cohor Study
Total and cause-specific mortality 2015	National and Subnational Burden of Diseases, Injuries, and Risk Factors in Iran (NASBOD)[3]
Type 2 diabetes incidence rate	Derakhshani et al study[4]

3-AZAR cohort profile:

The AZAR Cohort Study (azarcohort.tbzmed.ac.ir) is a prospective study assessing the risk factors related to the most prevalent NCDs in the province. More details about the Azar cohort study have been published elsewhere [5]. The inclusion criteria were: (i) being permanent residence in Shabestar district (minimum of 9 months); (ii) having written informed consent; (iii) having at least one Azeri parent; and (iv) being at the age range of 35 to 70 years, at the time of enrolling in the study. Exclusion criteria were: being diagnosed with (i) a disabling psychiatric disorder and (ii) being diagnosed with a disabling physical illness. The prevalence of type 2 diabetes was determined according to participants' self-report.

A Food Frequency Questionnaire (FFQ) assessment administered to the AZAR cohort participant to assess amount and frequency of food and drink consumption over the past year. This questionnaire includes 125 food items appropriate for the Iranian population, including bread, cereal, grains, meat and meat products, milk and dairy products, vegetables, fruits, types of oil and oilseeds, sugar, miscellaneous food products, spices and food supplements. Also, some local foods were added into the questionnaire. The nutrition questionnaire also asks about cooking methods, food preservation, food storage, cooking styles, and use of herbal medicines as well as drinks.

To help the respondent's memory and to enhance the accuracy and precision of participants' responses, household scales, including glasses, teaspoons, tablespoons, and colored photographs of portion size were used during the interview. All participants provided an answer to their frequencies of food intake (daily, weekly, monthly and annually) according to the standard portion size for each food item; then each participant's reported intake was transformed to weight using standard Iranian household measures. Dietary information was converted to energy and nutrients using USDA Food Composition Tables (USDA-FCT) to obtain daily energy intake of food items[6].

Table B- The mortality rate from Type 2 diabetes per 100,000 people in year 2015 in East Azerbaijan province

Age range(yr)	Female	Male	Both
30-34	0.95	1.39	1.17
35-39	1.36	1.75	1.56
40-44	2.63	2.57	2.60
45-49	5.38	5.85	5.62
50-54	9.45	11.82	10.61
55-59	19.87	19.11	19.49
60-64	36.02	35.93	35.98
65-69	68.29	66.50	67.48
70-74	107.22	71.62	90.59
75-79	178.41	130.19	154.71
80-84	226.43	138.19	184.02
+85	530.97	381.43	455.31

4-Model simulation

This model has been used to estimate the number of deaths prevented or delayed because of type 2 diabetes after implementing a specific policy. According to the methodology of a similar study

to obtain preventable or delayed death in the case of implementing a policy option in an age and sex group, the desired command code was written based on the following mathematical calculation and implemented in the software. As each policy option increases or decreases the amount of nutrients consumed, the effect of changes in the consumption on reducing or increasing the risk is calculated using the following formula:

$$1 - exp\left(log(RR) \times \left(\frac{B}{A}\right)\right)$$

Where, RR= Relative risk between SSB consumption level in baseline and type 2 diabetes extracted from meta-analyses; A=current amount of SSB consumed in the target state in terms of serving per day in the age and sex group; B= The amount of SSB consumption after implementing the desired policy option in terms of serving per day. The number obtained from the above formula is multiplied by the number of deaths due to type 2 diabetes while no policies are implemented. [7].

References:

1. Scarborough P HR, Mizdrak A, Zhou LM, Doherty A. . The preventable risk integrated ModEl and its use to estimate the health impact of public health policy scenarios. . *Scientifica.* 2014; 2014: 1-22.
2. Smed S SP, Rayner M, Jensen JD. . The effects of the Danish saturated fat tax on food and nutrient intake and modelled health outcomes: an econometric and comparative risk assessment evaluation. *European journal of clinical nutrition.* 2016 70(6): 681.
3. Peykari N, Hashemi H, Dinarvand R, Haji-Aghajani M, Malekzadeh R, Sadrolsadat A*, et al.* National action plan for non-communicable diseases prevention and control in Iran; a response to emerging epidemic. *Journal of Diabetes & Metabolic Disorders.* 2017; 16(1): 3.
4. Derakhshan A, Sardarinia M, Khalili D, Momenan AA, Azizi F, Hadaegh FJPo. Sex specific incidence rates of type 2 diabetes and its risk factors over 9 years of follow-up: Tehran Lipid and Glucose Study. 2014; 9(7): e102563.
5. Farhang S FE, Amini Sani N, Poustchi H, Ostadrahimi A, Alizadeh BZ, et al. . Cohort Profile: The AZAR cohort, a health-oriented research model in areas of major environmental change in Central Asia. *Inter J Epidemiol* 2019; 48: 382-.
6. Eghtesad S, Hekmatdoost A, Faramarzi E, Homayounfar R, Sharafkhah M, Hakimi H*, et al.* Validity and reproducibility of a food frequency questionnaire assessing food group intake in the PERSIAN Cohort Study. *Frontiers in Nutrition.* 2023; 10.

7. Pearson-Stuttard J, Bandosz P, Rehm CD, Penalvo J, Whitsel L, Gaziano T*, et al.* Reducing US cardiovascular disease burden and disparities through national and targeted dietary policies: A modelling study. *PLoS medicine.* 2017; 14(6).
